# Mitochondrial fatty acid beta-oxidation: a possible therapeutic target for skeletal muscle lipotoxicity in peripheral artery disease myopathy

**DOI:** 10.17179/excli2024-7004

**Published:** 2024-04-23

**Authors:** Cassandra E. Bradley, Emma Fletcher, Trevor Wilkinson, Andrew Ring, Lucas Ferrer, Dimitrios Miserlis, Pal Pacher, Panagiotis Koutakis

**Affiliations:** 1 Department of Biology, Baylor University, One Bear Place #97388, Waco, TX 76798, USA; 2 Department of Surgery, University of Texas at Austin Dell Medical School, 1601 Trinity St, Room 6708A, Austin, TX 78712, USA; 3 National Institutes of Health, Bethesda, MD, USA

**Keywords:** peripheral artery disease (PAD), mitochondria, age, ectopic lipids, fatty acid beta oxidation (FAO), acetyl coenzyme A carboxylase 2 (ACC2)

## Abstract

Peripheral artery disease (PAD) is an atherosclerotic disease impacting over 200 million individuals and the prevalence increases with age. PAD occurs when plaque builds up within the peripheral arteries, leading to reduced blood flow and oxygen supply to the outer extremities. Individuals who experience PAD suffer from ischemia, which is typically accompanied by significant damage to skeletal muscles. Additionally, this tissue damage affects mitochondria, causing them to become dysregulated and dysfunctional, resulting in decreased metabolic rates. As there is no known cure for PAD, researchers are exploring potential therapeutic targets by examining coexisting cardiovascular conditions and metabolic risk factors, such as the aging process. Among these comorbidities, type-two diabetes mellitus and obesity are particularly common in PAD cases. These conditions, along with aging itself, are associated with an elevated accumulation of ectopic lipids within skeletal muscles, similar to what is observed in PAD. Researchers have attempted to reduce excess lipid accumulation by increasing the rate of fatty acid beta oxidation. Manipulating acetyl coenzyme A carboxylase 2, a key regulatory protein of fatty acid beta oxidation, has been the primary focus of such research. When acetyl coenzyme A carboxylase 2 is inhibited, it interrupts the conversion of acetyl-CoA into malonyl-CoA, resulting in an increase in the rate of fatty acid beta oxidation. By utilizing samples from PAD patients and applying the pharmacological strategies developed for acetyl coenzyme A carboxylase 2 in diabetes and obesity to PAD, a potential new therapeutic avenue may emerge, offering hope for improved quality of life for individuals suffering from PAD.

## Introduction

Over 200 million individuals worldwide are afflicted by peripheral artery disease (PAD). Despite its substantial global impact, our understanding of the underlying pathology remains elusive (Fowkes et al., 2013[13]). Notably, aging, obesity, and diabetes mellitus have emerged as pivotal risk factors in the development of PAD (Hicks et al., 2018[20]; Shammas, 2007[43]). These factors predominantly affect individuals over 40, with an alarming 12-20 % of the global population experiencing PAD by the age of 65 (Song et al., 2019[45]). PAD, a form of arteriosclerosis, stems from the accumulation of plaque in the peripheral arteries, encompassing the lower extremities, as well as other vital areas like the coronary and cerebrovascular regions. PAD is a major cause of cardiovascular-related deaths (Kullo and Rooke, 2016[32]). Development of PAD is often accompanied by a spectrum of negative symptoms, including ischemic rest pain, claudication, critical limb ischemia, ulcers, gangrene formation in the lower extremities (leading to potential amputation), and possible death (Bonaca et al., 2021[3]; Choi et al., 2021[5]; Kim et al., 2020[26]). Plaque buildup can occur in any of the peripheral arteries of the legs or, less frequently, the arms of an individual as a variation of PAD (Kullo and Rooke, 2016[32]). The ultimate consequence of plaque buildup is the reduction of blood flow, subsequently diminishing the supply of oxygen to the extremities, resulting in progressive muscle deterioration (Pipinos et al., 2008[39]). Notably, compromised skeletal muscle integrity is closely associated with mitochondrial pathology (Pipinos et al., 2008[39]). As blood and muscle oxygen concentrations decrease, the mitochondria experience a scarcity of oxygen required for essential processes like oxidative phosphorylation (OXPHOS) and other vital metabolomic activities (Elzschig and Collard, 2004[10]). When the mitochondria have insufficient oxygen due to these ischemic conditions, the electron transport chain cannot produce adenosine triphosphate (ATP) energy and other crucial products indispensable for proper bodily functioning, including nicotinamide adenine dinucleotide phosphate (NADPH), flavin adenine dinucleotide (FADH_2_), and the subsequent electrochemical gradient (Stipanuk and Caudill, 2019[46]). Upon reperfusion events, oxygen is reintroduced to the muscle, resulting in the synthesis of reactive oxygen species (ROS) from hypoxanthine, which accumulates due to heightened rates of adenine nucleotide catabolism during prior ischemic episodes (Elzschig and Collard, 2004[10]). Notably, elevated ROS levels are not only linked to PAD but also to its known risk factors, such as aging (Schriner and Linford, 2006[42]). These ROS are toxic to the body and can cause further damage to the tissues and muscle cells via different mechanisms, including lipid peroxidation (Li and Jackson, 2002[33]).

As the mitochondria account for over 90 % of the oxygen consumed in skeletal muscles, which have dynamic and extensive energy needs, evaluating the pathology of skeletal muscle mitochondria can provide insights into the overall trajectory of the tissue's health (Kim et al., 2020[26]; Pizzimenti et al., 2019[40]). As disease progresses, the mitochondria associated with the skeletal muscle lose structural integrity both intrinsically and extrinsically, as the same myofibril structure is no longer present to anchor them in their proper orientation (Pipinos et al., 2008[39]). Sarcopenia, or age-related skeletal muscle deterioration, plays a role in progressing skeletal muscle damage in older individuals, which as previously stated, have an increased risk of PAD (Boengler et al., 2017[2]; Buford et al., 2010[4]; Goodpastor et al., 2006[16]). By itself, sarcopenia is associated with whole-body adiposity (Visser et al., 1998[51]), but overall skeletal muscle myopathy has also been linked to lipotoxic effects occurring in the body (Tamilarasan et al., 2012[50]). Lipotoxicity occurs due to an atypical accumulation of lipids in non-adipose tissue (Tamilarasan et al., 2012[50]). This accumulation of lipids can cause skeletal muscle damage coinciding with the previously mentioned change of structure (Tamilarasan et al., 2012[50]). Common abnormalities associated with altered/impaired skeletal muscle structure include mitochondrial dysfunction and fibers lacking centrally located mitochondria, which results in functional impairment and mobility loss (Koutakis et al., 2015[29][30]; Mietus et al., 2020[35]; McDermott et al., 2020[34]). When the mitochondria are disorganized, all metabolic processes, including the transport of macronutrients into the organelle, are impaired (Koutakis et al., 2014[31]; Pipinos et al., 2008[39]; Pizzimenti et al., 2019[40]). Even in less severe cases of PAD, structural impairment manifests as reduced muscle mass in the calf area, alongside increased fatty infiltration and fibrosis within the calf muscles. These changes can contribute to muscle stiffening, exacerbating the progression of damage (Mietus et al., 2020[35]; McDermott et al., 2020[34]). 

## Peripheral Artery Disease Treatments

At present, PAD remains without a known cure, regardless of its severity. One common intervention utilized to temporarily alleviate PAD symptoms is revascularization surgery. However, revascularization, while widely employed, is still an imperfect treatment approach, especially concerning the muscle aspect of PAD, which is not the primary focus of such surgical procedures (Ismaeel et al., 2018[24]; Koutakis et al., 2018[28]; Wu et al., 2021[55]). One of the most common forms of therapies regularly utilized by physicians to treat PAD is lifestyle modification therapy. These modifications tend to relate to the comorbid diseases typically associated with PAD. Comorbidities, like type-two diabetes mellitus, obesity, and other cardiovascular diseases, can also assist physicians in accurately diagnosing individuals with this disease (Shammas, 2007[43]). As previously mentioned, individuals with PAD may engage in behaviors linked to overall health risks. Therefore, altering one's diet, exercise regiment, and addressing smoking and alcohol consumption habits can significantly impact the disease's progression (Huang et al., 2017[22]). 

Other forms of therapies can also be used to combat the negative effects of PAD if life-style modification is insufficient, or there are patient compliance limitations. Some of these potential therapies include lipid-lowering, glucose-lowering, antithrombotic, and aspirin monotherapy (Bonaca et al., 2021[3]). Regrettably, many of these therapies show limited effectiveness in combating PAD (Bonaca et al., 2021[3]). For example, glucose-lowering medications effectively reduce associated risk factors but do not substantially ameliorate PAD itself (Bonaca et al., 2021[3]). Several more targeted pharmacological approaches are currently undergoing clinical trials. Among these emerging therapies are Metformin and Proprionyl-L-Carnitine (PLC). The primary goal of these clinical drug trials is to determine whether they improve a PAD patient's walking ability and/or synergize with other currently employed treatments, such as exercise (Khan et al., 2019[25]; NLM, NCT03054519, 2017[36]; Hiatt et al., 2011[19]). These pharmacological trials are exploring diverse molecular pathways within skeletal muscles, as the pathology of PAD muscles has not been attributed to a single centralized issue (Ismaeel et al., 2018[23]). Nevertheless, many of these potential drug therapies are designed to target pathways associated with mitochondria. As previously mentioned, mitochondrial damage plays a crucial role in the development, progression, and persistence of PAD in individuals. Therefore, a deeper exploration of the biological pathways related to proper mitochondrial functionality holds promise as a source of much-needed insight into potential specific therapeutic targets (Pizzimenti et al., 2019[40]).

## Mitochondria Functionality in the Presence of Lipids

As mentioned previously, mitochondria serve as a significant source of ROS production during regular metabolic processes, and mitochondrial damage and the subsequent decline in organ function, is a part of the aging process (Schriner and Linford, 2006[42]). Excessive ROS accumulation can lead to cell death, organ failure, various diseases, and ultimately death (Beckman and Ames, 1998[1]). However, despite numerous longevity studies centered around ROS production, no promising results have emerged. This suggests that ROS play a complex role in aging and age-related diseases and that focusing on interrelated processes and pathways may result in better understanding of the pathology (Stuart et al., 2014[47]). When mitochondria function optimally, multiple metabolic pathways are maintained in homeostasis, including the glycolytic pathway, beta-oxidation, the Krebs cycle, and fatty acid (FA) synthesis. However, individuals with PAD tend to exhibit a higher concentration of ectopic lipids accumulating in the skeletal muscle, as is also seen in individuals with type-two diabetes, obesity, and the elderly (Olzmann and Carvalho, 2019[38]; Walther and Farese, 2012[53]; Zoico et al., 2010[57]). This lipid buildup can create a lipotoxic environment (Tamilarasan et al., 2012[50]). Within skeletal muscle cells, lipid droplets (LDs) are considered one of the primary storage sites for lipids (Olzmann and Carvalho, 2019[38]; Walther and Farese, 2012[53]). LDs house intramuscular triglycerides (IMTGs) which are transformed into intramyocellular lipids (IMCLs) during times of metabolic stress (Coen and Goodpaster, 2012[6]). They play essential roles in satellite cell homeostasis, tissue regeneration, and determining the fate of stem cells with respect to their metabolic role (Olzmann and Carvalho, 2019[38]). In individuals with PAD, LDs are more abundant and larger, reflecting the need for increased storage of ectopic lipids they accumulate. These bulkier LDs could potentially serve as therapeutic targets for addressing skeletal muscle disorganization in PAD due to elevated lipid levels and are a source of untapped energy reserves (Daemen et al., 2018[7]). Additionally, elevated levels of fatty acids (FAs) in both adipose and muscle tissue due to lipid accumulation can activate certain metabolic pathways at higher rates than usual, particularly beta-oxidation (Wajner and Amaral, 2016[52]). As an alternative to the glycolytic pathway, the body can utilize long-chain FAs to generate ATP energy through mitochondrial fatty acid β-oxidation (FAO) (Yan, 2015[56]).

## Fatty Acid β-Oxidation

FAs are typically stored in adipose tissue as triglyceride (TG) molecules when there is no immediate need for them in the body (Drosatos and Schulze, 2013[9]). These TG reserves are tapped into during metabolic stress periods, including extended fasting or starvation, strenuous exercise, infection, and more (Wajner and Amaral, 2016[52]). The process of mobilizing and converting TGs into FAs through fatty acid oxidation (FAO) relies on the interaction of various lipolytic proteins, collectively forming a 'lipolytic complex.' This complex comprises multiple hormone-sensitive lipase (HSL) isoforms, monoacylglycerol lipase (MGL), adipocyte lipid binding protein (ALBP), among others (Haemmerle et al, 2003[18]; Recazens et al, 2021[41]). Over 25 proteins are suggested to work together to ensure efficient FAO (Wajner and Amaral, 2016[52]). HSL is known for its ability to hydrolyze various lipid substrates beyond triglycerides, giving it a broad range of specificity and applications (Donsmark et al., 2004[8]; Recazens et al., 2021[41]). However, the exact mechanisms underlying HSL-induced lipolysis remain incompletely understood and require further research. Importantly, insulin can dephosphorylate HSL, leading to the inhibition of FAO, suggesting impaired FA hydrolysis in individuals with diabetes and obesity, underscoring HSL's pivotal role in this process (Donsmark et al., 2004[8]; Haemmerle et al, 2003[18]).

Biologists have debated the benefits and disadvantages of increased rates of FAO in skeletal muscle tissue. However, multiple studies suggest that it is not the increase of FAO that can cause problems, like myopathies, in both skeletal and cardiac muscle, but it is the inability of the body to keep up with FAO demands in response to elevated levels of lipids resulting in lipotoxic effects and ectopic build up (Houten et al., 2016[21]; Wajner and Amaral, 2016[52]). Altering the rate at which mitochondria engage in FAO and establishing a preference for this metabolic pathway could potentially serve as a therapeutic approach for individuals with excess FA buildup. As mentioned earlier, individuals with PAD often have comorbidities like obesity and type-two diabetes, which could contribute to the development of this disease (Kullo and Rooke, 2016[32]). It is plausible that regulating the FAO pathway specifically in skeletal muscle cells, as suggested by previous studies involving the manipulation of regulatory steps and FAs, might offer a means of therapy for PAD (Gautam et al., 2014[14]; Stipanuk and Caudill, 2019[46]; Wajner and Amaral, 2016[52]). Enhancing FAO rates could help mitigate the adverse effects of FA accumulation associated with lipotoxicity. Notably, mitochondrial dysfunction, whether in isolation or in tandem with transporter damage, significantly influences the development and progression of PAD. Therefore, by focusing on this pivotal organelle, a multitude of potential therapeutic avenues can be explored (Pipinos et al., 2008[39]).

## The FAO Mechanism

**Figure 1:**
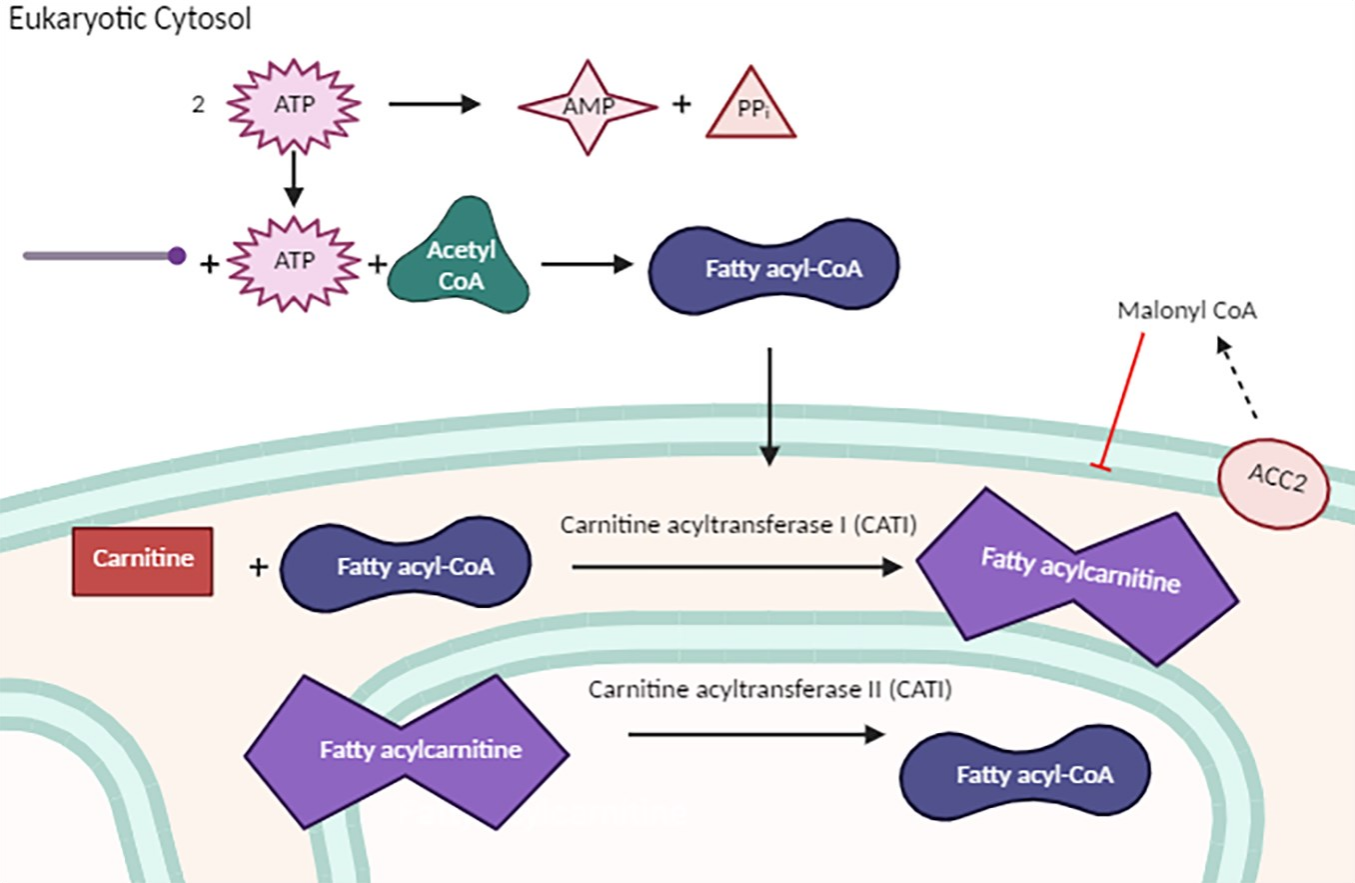
*Mitochondrial Fatty Acid Transport*. A visual representation of the base pathway involved in moving long chain FAs into the inner mitochondrial membrane via the carnitine pathway. This process begins when a long chain FA (represented by the round-headed line) reacts with a single acetyl CoA and ATP molecule creating fatty acyl-CoA within the eukaryotic cytosol. Fatty acyl-CoA then enters the mitochondrial outer membrane to react with carnitine, with CAT1 as a catalyst, resulting in fatty acylcarnitine. Fatty acylcarnitine is then able to permeate the inner mitochondrial membrane to react with CAT2 to be converted back into fatty acyl-CoA.

**Figure 2:**
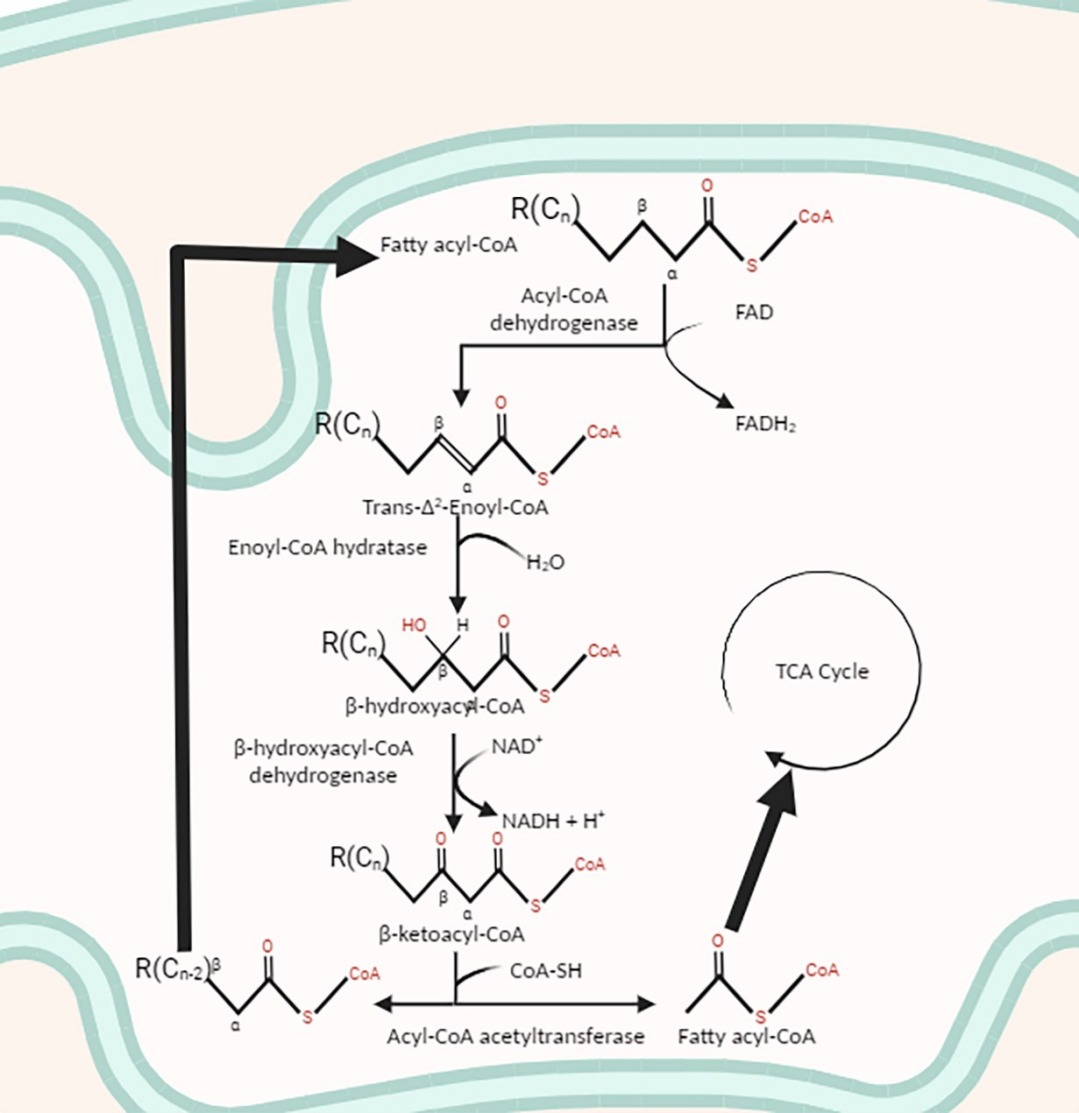
*Catabolism of Fatty Acids in the Mitochondria via Beta-Oxidation*. A visual representation of the base pathway involved in catabolizing the fatty acyl-CoA derived from long-chain FAs. Once the Fatty acyl-CoA enters the inner mitochondrial matrix the beta-carbon on the chain is oxidized in four separate steps resulting in the removal of two carbons creating an acetyl-CoA and a shorter-chained fatty acyl-CoA. The new fatty acyl-CoA will be processed through beta-oxidation until it no longer can, and the resulting acetyl-CoA will be used to create ketone bodies, synthesize FAs, or enter the TCA cycle to produce energy to suit the organism's needs.

The FAO process, which takes place exclusively within mitochondria, is far from a straightforward linear pathway. FAO varies significantly depending on numerous factors, including but not limited to the length, saturation, and evenness in the carbon number of the fatty acid chain (Stipanuk and Caudill, 2019[46]). To undergo FAO, fatty acids must first traverse the mitochondrial membrane barrier through a series of transformative steps. Typically, when a saturated even-chain fatty acid encounters ATP (which is cleaved by pyrophosphatase to form AMP and PPi) and CoA, it gives rise to fatty acyl-CoA in the cytosol (Figure 1(Fig. 1)) (Stipanuk and Caudill, 2019[46]). The carnitine transportation system then comes into play, involving both carnitine acyltransferase I (CATI) and carnitine acyltransferase II (CATII) (Stipanuk and Caudill, 2019[46]). After transportation through the outer mitochondrial membrane, the fatty acyl-CoA interacts with carnitine, forming fatty acylcarnitine. This compound is transported across the inner mitochondrial membrane by CATI, also known as carnitine palmitoyl transferase I (CPTI) (Stipanuk and Caudill, 2019[46]). Once the fatty acylcarnitine enters the mitochondrial matrix, aided by CATII or carnitine palmitoyl transferase II (CPTII), it initiates beta-oxidation and subsequently enters the Krebs cycle (Figure 2(Fig. 2)) (Stipanuk and Caudill, 2019[46]). Unsaturated fatty acids (UFAs) follow a different metabolic pathway depending on factors such as the number of carbons, whether the carbons are in even or odd numbers, and the location of the unsaturated double bond (Stipanuk and Caudill, 2019[46]). FAO of UFAs continues until the unsaturated double bond is near the carboxyl end of the molecule, at which point the process halts. Overall, the net energy gain from this process hinges on the specific fatty acid introduced into the mechanism and its chemical properties. Nevertheless, it is widely acknowledged that the net ATP yield from FAO is generally higher than what is produced in a single glycolytic cycle (Stipanuk and Caudill, 2019[46]). 

In addition to the base components that make up FAO, there are a plethora of enzymes that act on the pathway in both excitatory and inhibitory manners. One of the proteins that has been suggested to inhibit the beta-oxidation pathway is malonyl-CoA. Malonyl-CoA acts as part of a negative feedback loop acting on CATI in an inhibitory fashion (Stipanuk and Caudill, 2019[46]). The production of malonyl-CoA from acetyl-CoA is triggered by the presence of the acetyl coenzyme A carboxylase 2 (ACC2) protein localized in the outer mitochondrial membrane in eukaryotes (Stipanuk and Caudill, 2019[46]). AMP-activated protein kinase (AMPK), which can be activated by ischemic events, phosphorylates ACC causing ACC to be inhibited (Folmes and Lopaschuk, 2007[12]). This inhibition of ACC can limit the synthesis of malonyl-CoA resulting in a decrease of CATI activity ultimately increasing FAO rates (Folmes and Lopaschuk, 2007[12]). Both isoforms of acetyl coenzyme A carboxylase, ACC1 and ACC2, have been recognized for their substantial roles in cellular metabolism, extending to both prokaryotes and eukaryotes. Acetyl coenzyme A carboxylase 1 (ACC1) is primarily located in the cytoplasm of eukaryotic cells and assumes a pivotal role in fatty acid synthesis. ACC1 also facilitates the conversion of acetyl-CoA into malonyl-CoA and is typically localized in lipogenic tissues (Wang et al., 2022[54]). In contrast, ACC2 predominantly accumulates in oxidative tissues, including cardiac and skeletal muscle (Wang et al., 2022[54]). The presence of ACC2 in oxidative tissues has led scientists to conclude that ACC2 plays a critical role in cardiomyopathies by influencing the rate of FAO (Shao et al., 2020[44]). 

Cardiomyopathies represent a group of heart diseases characterized by the accumulation of damaged and dysfunctional mitochondria in cardiac muscle, a condition that can arise when mitophagy is dysregulated. Researchers have demonstrated ACC2's significance through studies involving mice with obesity and diabetes that were genetically modified to have a knockout of the genes encoding the known ACC2 regulator. This genetic alteration increased the activation rate of FAO (Kolwicz, 2012[27]). The resulting rise in FAO rates was associated with a decrease in high-fat diet-induced cardiac dysfunction, as observed by scientists (Shao et al., 2020[44]).

## Acetyl Coenzyme A Carboxylase

Scientific investigations into the deletion of acetyl coenzyme A carboxylases (ACCs) have taken various approaches, recognizing the presence of two distinct isomers in eukaryotes. These experiments have emphasized the need for specificity when targeting ACC isomers to avoid unintended side effects (Olson et al., 2010[37]). Early research involved the simultaneous knockout of both ACC1 and ACC2, impacting overall fatty acid oxidation (FAO) rates due to the distinct localization and activity of these two isomers. The results of these initial studies supported the use of ACC inhibitors as potential treatments for diabetes (Olson et al., 2010[37]). Subsequent research focused on individually knocking out the isomers. Kolwicz et al. (2012[27]) successfully developed a cardiac-specific ACC2 deletion, demonstrating its impact on systemic metabolism. The metabolic changes observed resulted in an impairment of bioenergetics as well as function, as ACC2 deletion prevented these alterations from occurring during a state of pressure-overload (Kolwicz et al., 2012[27]). Notably, this work emphasized that an increased rate of FAO does not detrimentally affect the heart. Instead, an imbalance between lipid supply and oxidation rate plays a role in myocardial energetic dysfunction, challenging the notion that increased FAO produces more reactive oxygen species (ROS) and subsequent damage (Kolwicz et al., 2012[27]). However, it remained unclear whether mechanistic dysfunction was a cause or consequence of this metabolic imbalance.

To further explore the molecular mechanisms underlying lipotoxic cardiomyopathies, Shao et al. (2020[44]) introduced an ACC2-specific genetic knockout into an obese mouse model. This genetic alteration protected obese mice from cardiac dysfunction and associated negative consequences (Shao et al., 2020[44]). FAO increased without adverse effects, preventing cardiomyopathies typically associated with obesity. Cardiac energetics were maintained, lipid accumulation in the heart was prevented, mitochondrial dysfunction resulting from obesity was averted, damaged mitochondria accumulation was reduced, and mitophagy rates were restored (Shao et al., 2020[44]). The study highlighted the role of parkin, a protein involved in ubiquitination and mitophagy regulation, in maintaining mitophagy homeostasis. High lipid levels reduced parkin expression, leading to decreased mitophagy rates, but this effect was reversed when FAO rates were increased (Shao et al., 2020[44]). Importantly, these findings have implications for PAD research, as individuals with PAD and comorbidities often experience cardiomyopathy events. While ACC2 genetic knockouts have shown improvements in the FAO pathway without negative side effects, some studies have presented conflicting results (Olson et al., 2010[37]). Given the existence of two ACC isomers, genetic manipulation may inadvertently alter ACC1, affecting tissues beyond the muscle. Researchers like Glund et al. (2012[15]) and Takagi et al. (2020[49] and 2021[48]) have been able to circumvent the inconsistencies present in the results of genetic manipulation using a pharmacological approach (Griffith et al., 2014[17]). Glund et al. (2012[15]) administered (s)-9c, a selective inhibitor of ACC2, orally to obese mice with type-two diabetes and measured both their weight change as well as glucose levels over a 70-day period. The treatment resulted in glucose-lowering effects in both blood and muscle, both in the short- and long-term, with other positive effects emerging within 3-4 days of treatment (Glund et al., 2012[15]). Since lowered blood glucose often leads to increased FA utilization, such pharmacological strategies to enhance FAO rates could serve as a potential therapeutic avenue for PAD by addressing ectopic lipid accumulation. Takagi et al. (2020[49] and 2021[48]) utilized a different selective ACC2 inhibitor, compound 2e, for short and long-term studies in the skeletal muscle of mice with type-two diabetes. This research resulted in short-term and long-term reductions of diacylglycerol and ceramide leading to improved whole-body insulin resistance and hyperglycemia as well as reducing diabetes progression (Takagi et al., 2020[49], 2021[48]). This work conducted in diabetic mice with (s)-9c and compound 2e could be replicated in an aging murine model previously shown to emulate PAD myopathy (Fletcher et al., 2023[11]).

## Conclusion

Considering the limited availability of highly effective therapies for treating PAD myopathy, it is of paramount importance to intensify our research into the underlying pathogenic mechanisms. PAD is a prevalent age-related disease that can lead to devastating outcomes for affected individuals (Kullo and Rooke, 2016[32]). Given that mitochondria play a pivotal role in maintaining the homeostasis and structural integrity of skeletal muscle cells, gaining a deeper understanding of the metabolic processes governed by mitochondria, especially the interplay between FAO and muscle structural damage, is imperative (Pipinos et al., 2008[39]; Pizzimenti et al., 2019[40]). Considering the elevated levels of lipotoxicity observed in cells of individuals with PAD myopathy, the toxic effects of these fatty acids through the promotion of FAO. One potential avenue to enhance the activation of this pathway is through pharmacological interventions, including drugs targeting and deactivating ACC2. Selective ACC2 inhibition has the potential to emerge as a novel therapeutic approach aimed at preventing the infiltration and accumulation of fatty acids within skeletal muscles. Ultimately, this singular pathway at the subcellular organelle level could hold the key to establishing an innovative treatment with a significant impact on PAD patients.

## Declaration

### Competing interests

The authors have no competing interests.

### Acknowledgments

All figures were created with BioRender.com. This work was supported by Baylor University.
